# Just allocation of COVID-19 vaccines

**DOI:** 10.1136/bmjgh-2020-004812

**Published:** 2021-02-13

**Authors:** Anders Herlitz, Zohar Lederman, Jennifer Miller, Marc Fleurbaey, Sridhar Venkatapuram, Caesar Atuire, Lisa Eckenwiler, Nicole Hassoun

**Affiliations:** 1 Institute for Futures Studies, Stockholm, Sweden; 2 Rambam Health Care Campus, Haifa, Israel; 3 School of Medicine, Yale University, New Haven, Connecticut, USA; 4 Princeton University, Princeton, New Jersey, USA; 5 King's College London, London, UK; 6 University of Ghana, Legon, Greater Accra, Ghana; 7 George Mason University, Fairfax, Virginia, USA; 8 Binghamton University, Binghamton, New York, USA; 9 Cornell University, Ithaca, NY, USA

**Keywords:** public health, immunisation

Authorized COVID-19 vaccines must be distributed fairly. Several proposals have emerged offering guidelines for how to do this. However, insofar as the aim is to have the greatest health impact, these proposals fall short. We offer three suggestions to strengthen them

The most advanced attempt at coordinating vaccine distribution is the COVID-19 Vaccines Global Access (COVAX) facility, a collaboration that brings together governments, companies, international organisations and others to accelerate the development and manufacture of COVID-19 vaccines.^1^ A total of 182 countries have joined the facility so far, which has secured about US$2 billion for its advance market commitment (AMC). The AMC will allow 92 low-income and middle-income countries to obtain vaccine doses as they are approved or authorised. Currently, COVAX is set up so that in a first phase poor countries can vaccinate 3% of their populations, while rich countries can vaccinate up to 50%. Though the facility hopes to allow all members to vaccinate at least 20% of their populations by the end of 2021.^2 3^ Other proposals by the WHO suggest prioritising healthcare workers, the elderly and, those with comorbidities that put them at greater risk of severe illness if infected with COVID-19, people from certain high-risk sociodemographic groups and some teachers.

Another sophisticated proposal, the ‘Fair Priority’ Model, suggests countries with vaccines contribute to global distribution once their COVID-19 transmission rates drop to R<1. In phase I, they argue that vaccines should primarily be distributed to minimise standard expected years of lives lost. Other considerations, such as gross national income and poverty rates, can and should become prominent in later stages of distribution. Finally, researchers at Vanderbilt University suggest distributing vaccines to countries based on their ability to distribute vaccines, their capacities to provide care and whether they have helped test and develop new interventions.^4^

These proposals, while helpful, have three critical limitations. First, they fail to recognise that fairness should primarily concern mediation of claims and interests of different persons, not countries. Proposals for fair distribution must, in other words, address health problems for individuals. Moreover, since most individuals have little choice as to their country of origin or residence, we should not discriminate against them based on location. A fair proposal cannot allow rich countries to hoard vaccines or prioritise their own populations first, nor can it give individuals less priority simply because they live in a country with less infrastructure, capacity or willingness to distribute vaccines. Finally, equity between individuals in achieving the greatest health impact is probably not compatible with equal distribution on the country level. We expand on some of these points below.

Second, allocation principles must explicitly focus on both direct and indirect health effects of COVID-19. Direct health effects include death and disability caused (in full or in part) by the virus. Indirect health effects include death and disability caused (in full or in part) by the social response to the virus. One major concern, for instance, is how COVID-19 indirectly will have devastating consequences in India and other low- and middle- income countries since the response to the pandemic undermines existing infrastructure to manage other infectious diseases such as malaria, tuberculosis and HIV. A vaccine allocation policy based on a desire to mitigate the negative health impact of the pandemic must explicitly aim to reduce all mortality and morbidity, not just morbidity and mortality directly attributable to COVID-19.

Third, having the greatest global health impact requires assisting countries with their vaccine distribution, production and consumption. A fair allocation system must consider how vaccine distribution will determine the success of whatever strategy is adopted. Vaccines may differ significantly in their effectiveness and the resources they require for successful and wide distribution and consumption. Some vaccines, such as Pfizer’s mRNA vaccine, need to be stored at extremely low temperatures (−70°C). This makes this vaccine highly impractical in many countries where adequate transportation networks, consistent energy supplies and sufficient cold chain storage are lacking (in only 28% of sub-Saharan Africa do healthcare facilities have reliable energy).^5^ Many vaccines also require multiple injections over time, which make them difficult to distribute, especially in countries that lack the staff to administer the vaccines or where the nearest clinic is a multiple days’ walk away. This means streamlining distribution chains from vaccine producer to vaccine consumer and producing and distributing auxiliary equipment (eg, bottles for vaccine, syringes, distribution clinics).

Efforts must also be geared towards maximising vaccine production. Currently, at least 80% of manufacturing capacity is in the generics sector.^6^ This sector is an extremely valuable resource that should be included in a responsible response to the pandemic. This may be done in different ways, for example, collaborations between vaccine developers and actors in the generics sector.^7^

Additionally, policy-makers should put in place measures to promote impactful uptake of vaccines rather than their mere distribution. Ensuring transparent, accurate and trustworthy information about vaccines can help advance uptake.^8–11^ Manufacturers should consider full clinical transparency, that is, sharing trial protocols and timely results dissemination and sharing of individual patient-level data to bolster public confidence in an approved vaccine. What is more, working to understand people’s particular and often legitimate reasons for distrust that go beyond incomplete or distorted information, like long-term economic hardship or past exploitation of a given community by scientific and medical institutions, and working to create the social, economic and political conditions that would enable more trusting relationships is crucial. Indeed, we must recognise trust and trustworthiness as global health goods that must be in place prior to the arrival of biomedical interventions.^12^

SARS-CoV-2 has been catastrophic in rich countries as well as in poor ones, but a fair vaccine allocation must help us combat the pandemic’s direct and indirect health effects for individuals irrespective of country of origin or residence. We cannot allow rich countries to prioritise their own populations. Furthermore, we should focus well beyond COVID-19’s direct health effects and enhance vaccine production, distribution and uptake. Endorsing the WHO’s Solidarity Call to Action for equitable global access to COVID-19 health technologies can help everyone secure safe and effective vaccines for COVID-19 as soon as possible (16). In a global pandemic, an outbreak anywhere threatens people everywhere.

## Data Availability

All data relevant to the study are included in the article.
